# Light-Tuned DC Conductance of Anatase TiO_2_ Nanotubular Arrays: Features of Long-Range Charge Transport

**DOI:** 10.3390/nano8110915

**Published:** 2018-11-07

**Authors:** Dmitry A. Zimnyakov, Michail Yu. Vasilkov, Sergey A. Yuvchenko, Alexey S. Varezhnikov, Martin Sommer, Victor V. Sysoev

**Affiliations:** 1 Physics Department, Yuri Gagarin State Technical University of Saratov, 77 Polytechnicheskaya str., Saratov 410054, Russia; vasilk.mikhail@yandex.ru (M.Y.V.); yuv-sergej@yandex.ru (S.A.Y.); alexspb88@mail.ru (A.S.V.); vsysoev@sstu.ru (V.V.S.); 2 Precision Mechanics and Control Institute of Russian Academy of Sciences, 24 Rabochayastr., Saratov 410024, Russia; 3 Saratov Branch of Kotelnikov Institute of Radio-Engineering and Electronics of Russian Academy of Sciences, 38 Zelenaya str., Saratov 410019, Russia; 4 Institute of Microstructure Technology, Karlsruhe Institute of Technology, 1 Hermann-von-Helmholtz Platz, 76344 Eggenstein-Leopoldshafen, Germany; martin.sommer@kit.edu

**Keywords:** anatase nanotubes, dc conductance, laser irradiation, inter-band transition, Urbach energy, charge mobility, drift current, diffusion current

## Abstract

Experimental results related to the photoactivated dc conductance of anatase TiO_2_ nanotubular arrays (aTNTAs) under pulsed irradiation by a laser light inside and outside the fundamental absorption band are presented. It is found that the mobility and diffusion coefficients of charge carriers in the examined aTNTA are extremely low due to a strong charge-phonon coupling, abundance of shallow traps, and hopping conductivity between adjacent nanotubes. We consider that the confining electric field appeared within the array structure due to the difference in the local concentrations of excess electrons and holes at large values of the dc conductance suppresses the drift current. In this case, the dc conductance of such aTNTAs is mainly matured by the diffusion of mobile carriers. A recurrent kinetic model for evolution of the dc conductance of aTNTAs under laser irradiation has been proposed to interpret the experimental results.

## 1. Introduction

Over the past three decades, titania-based nanostructures have been studied intensively as promising platforms in the various areas of material science and related technologies [1]. Rather important applications include photovoltaics [2] and photocatalytic chemistry [3], which originate from an interaction between the light (electromagnetic irradiation) and the given material. In the latter case, an extremely high photocatalytic activity of the UV-irradiated anatase and rutile TiO_2_ nanophase stimulated the appearance of a great number of theoretical and experimental works devoted to various aspects of a photo-induced charge transfer in the bulk and nanostructured titanium dioxide (see, e.g., References [4,5,6,7,8,9,10,11,12]). However, despite the abundance of these works, all the possible mechanisms of charge transfer, coupling, decoupling, trapping, and release from the traps in the photo-activated titania nanostructures are still far from complete understanding. In particular, the lifetime values reported in the various works for photo-generated mobile carriers in the micro- and nanostructured anatase vary from tens of picoseconds to even seconds [8,9,10,11]. In addition, these nanomaterials often exhibit very low carrier mobility in comparison with other wide-band semiconductors. These features are particularly due to the significant influence of mobility-limiting effects, such as strong phonon-charge coupling, and abundance of bulk and surface shallow traps, etc., on the charge transfer within these materials (see, for instance, References [13,14]). On the other hand, the act of final capture of slowly wandering carriers by the deep surface traps leads to increasing photocatalytic activity in the system [15,16].

Recently, peculiarities of the generation of carriers and their transfer found a great interest in the chemical sensing where light irradiation could particularly reduce the energy consumption by gas sensors [17,18,19]. The primary interest is to find options for the sensor operation at room temperature without a loss in high sensing capacity [20,21,22]. Here, the simplest way to create a chemiresistive layer between the flat metal electrodes is sedimentation of oxide nanostructures over the inter-electrode gap. Normally, the following two aspects are addressed: the characteristic geometrical size of the structures should be in the nanodomain in order to match the Debye length; and the overall layer should have a bigger surface area for gas access from the surroundings. Therefore, quasi-1D or quasi-2-D structures like nanoplatelets, nanorods or nanobelts, are considered as the most promising ones [23,24]. Among these structures, anatase titanium dioxide nanotube arrays (aTNTAs) have high potential [25,26]. Further adjusting chemiresistive characteristics of such layers via tuning their electrical properties by introducing a photoactivation control are of higher interest. Therefore, the goal of this work is to study the possibility of photoactivation-based engineering of the direct-current (dc) conductance in the case of partially ordered assemblies of densely packed anatase TNTAs. In addition, this study can be useful for better understanding the physical mechanisms of charge carrier transfer in the anatase-based nanostructured materials.

## 2. Experimental Techniques and Results

### 2.1. Synthesis and Characterization of Partially Ordered Assemblies of Densely Packed aTNTAs

**Figure 1:**
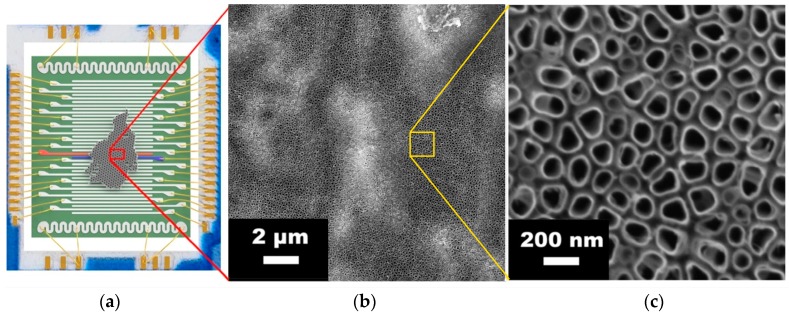
The multielectrode chip equipped with aTNTA layer: (**a**) cartoon of the studied chip with a piece of aTNTA membrane; red and blue electrodes indicate the layer segment under investigation; (**b**,**c**) the SEM images of the layer surface under different magnifications.

The aTNTA layer has been fabricated by electrochemical anodization of the titanium foil 0.127 mm thick (99.7%, Sigma-Aldrich, St. Louis, MO, USA), according to the earlier reported protocols [27]. In brief, the process has been carried out at constant electrical bias of 30 V for approx. 1 h. The oxidized foil has been etched in Br_2_ (ACS reagent, Sigma, USA) and CH_3_OH (abs., Emsure, Merck, Kenilworth, NJ, USA) mixture with the volume ratio of the components of 1:9 for 2 h at room temperature in order to remove the remains of titanium substrate according to the procedure described by Albu et al. [28]. The obtained aTNTA membrane has been placed into deionized water. Then, the membrane is drawn from the surface onto the chip equipped with the multiple Pt electrodes of 4 mm length, which is described in Reference [29]. The membrane connects the number of electrodes used to measure the aTNTA layer local resistance (Figure 1a). The distance between the two adjacent electrodes is approximately 90 μm. The aTNTA layer located between each pair of electrodes is considered as a resistive segment. All the electrodes have been ultrasonically wired to the ceramic card equipped with Erni 50-pin socket. Finally, the prepared chip has been the subject of drying and annealing at 300 °C in the air atmosphere for approximately 24 h. to finalize the aTNTAs crystallization.

The morphology of the aTNTA layer has been inspected by scanning electron microscopy (SEM) with the help of a two-beam workstation AURIGA^®^ Crossbeam (Carl Zeiss Group, Oberkochen, Germany) at 5–20 kV of acceleration voltage (Ga cathode). As shown in Figure 1b, the layer is continuous with a low number of defect areas mostly consisting of a compact oxide. The latter appears during the anodization process due to the oxygen arisen bubblers which may cover some foil spots and reduce their dissolution. However, a bigger part of the surface contains partially ordered tightly-packed nanotubes with double-sided shells having irregular circle shapes (Figure 1c). Some brighter areas observed in Figure 1b relate to spatial distortions of the aTNTA [27]. The appeared nanotubes stand perpendicular to the substrate. The average inner diameter of the pores inside the nanotubes varies within 108 ± 3.4 nm while the average wall thickness is 12.8 ± 0.7 nm. The height of the nanotubes does not exceed 1 μm. The average surface density of aTNTAs is about 4 × 10^9^ cm^−2^. Accounting for this geometry, the relative titanium dioxide content is about 0.2 of the total volume of the given nanoporous layer.

Previously we have reported [27] that the aTNTAs consist of polycrystals with the anatase TiO_2_ structure having high non-stoichiometry due to the existence of pristine defects like oxygen vacancies which are primarily responsible for available electron concentration, and, hence, for electrical properties of this material. The crystallite size in the walls of aTNTAs exceeds 25 nm (see the Supplementary, Figure S1) in accordance with other published investigations [30,31,32,33].

Following the aTNTA layer placing over Pt electrodes in the chip, we have checked the electrical contact at the layer/electrode interface at room temperature by I-V measurements. The typical I-V dependence measured by changing the voltage from −10 V to +10 V and vice versa has been found to follow the *I~U*^1.8^ curve. Such non-linearity primarily appears due to a fundamental difference between the work functions of Pt (Φ = 5.8 eV) and TiO_2_ (110) (Φ = 5.3 eV) [34] that make the pristine Schottky barrier for electron transfer. Also, no special care has been taken in these samples to introduce Pt ions into the aTNTAs as the deposition of electrodes over the aTNTA layer, which ordinarily reduces the interface space charge region. Additionally, there are potential barriers both between the grains in the aTNTA polycrystalline structure and anatase nanotubes themselves. All of that makes I-V curves non-linear under the given conditions. The typical values of total resistance measured for all the segments of the aTNTA layer located between the electrodes have been in the GOhm range that required special care to measure their conductance.

### 2.2. Measurements of the Photo-Induced DC Conductance of aTNTA

**Figure 2:**
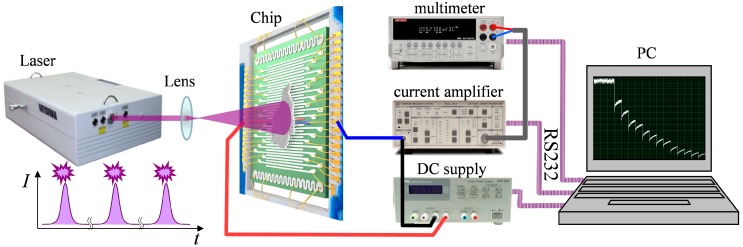
The scheme of experimental setup to measure the dc conductance of the aTNTA layer under laser irradiation in the range from 355 to 575 nm.

In order to perform activation of electrical properties of aTNTA by the visible-to-UV irradiation, we have developed an experimental setup drawn in Figure 2. The setup includes two major parts which are dealing with electrical and optical measurements. The electrical part has been designed in order to measure resistance of the aTNTA layer between a couple of electrodes by applying dc voltage and measuring the current magnitude in the two-probe mode. Because the primary resistance of the aTNTA layer is rather high, we have employed a high-gain, up to 1 pA/V, current amplifier (SRS-570). The pre-amplified current has been measured by the multimeter (Keithley 2000), and following the analog-to-digital conversion, has been passed to PC via the RS232 interface for storage and processing by the home-made software. The measuring voltage has been installed equal to 5 V.

A wavelength-tunable parametric computer-aided laser system (LS 2145 OPO type, Lotis TII) was applied as a source of probe light. The system provided the following irradiation parameters: the pulse duration is 10 ns, the wavelength range is 355–575 nm, the pulse repetition rate is from 10 Hz to 0.01 Hz, the maximal pulse energy at the minimal applied wavelength (355 nm) is 70 mJ, the output beam diameter is 6.3 mm, and instability of the pulse energy is less than 10% for all irradiation modes. The output pulse energy was measured using the light energy/power meter Gentec Maestro with a beam splitter (these units are not shown in Figure 2).

The analyzed segment of the aTNTA was irradiated by a diverging laser beam formed using the convex lens with a short focal length (38 mm). The distance between the irradiated sample and the beam waist was equal to 500 mm, and the diameter of the light spot on the sample surface was equal to 100 mm. The preliminary analysis of energy density distribution across the light spot has shown the near-Gaussian form. Correspondingly, we can conclude that non-uniformity of the energy fluence across the area of irradiated segment of aTNTA did not exceed 5%.

**Figure 3:**
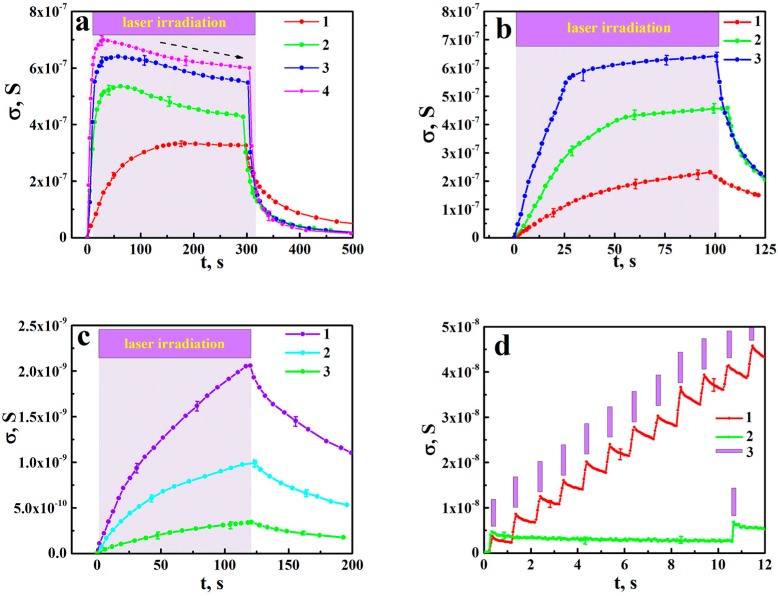
The experimental data on the temporal evolution of the dc conductance of aTNTA under laser irradiation: (**a**–**c**) long-term behavior of conductance under the action of pulse sequences, (**a**) the fixed repetition rate (10 Hz) and wavelength (355 nm) with varied irradiation fluence (1—0.1 mJ/cm^2^, 2—1.0 mJ/cm^2^, 3—2.2 mJ/cm^2^, 4—4.0 mJ/cm^2^); (**b**) the fixed irradiation fluence (3.5 mJ/cm^2^) and wavelength (355 nm) with the varied repetition rate (1—2 Hz, 2—6 Hz, 3—10 Hz); (**c**) the fixed irradiation fluence (3.5 mJ/cm^2^) and repetition rate (10 Hz) with the varied wavelength (1—425 nm, 2—475 nm, 3—525 nm); selectively shown error bars correspond to the significance level of 0.9; (**d**) short-term behavior under irradiation at low repetition rates (1—1 Hz; 2—0.1 Hz; 3—the moments of pulse laser irradiation On); irradiation fluence is 3.5 mJ/cm^2^ and the wavelength is 355 nm.

We have examined the chosen segment of the aTNTA placed between two Pt electrodes in the extensive course of photo-activation treatment under widely varying photo-activation parameters such as laser pulse energy, repetition rate of laser pulses and wavelength of acting radiation. The typical results are summarized in Figure 3a–c to plot the values of the dc conductance against irradiation time under various values of pulse energy, repetition rate and wavelength. In addition, short-term responses of the analyzed aTNTA segment under low repetition rates of laser pulses are shown in Figure 3d.

Additionally, we have carried out the measurements of the photoluminescence (PL) effect in the investigated aTNTA in the case of their photo-activation at 355 nm. The expected photoluminescence has been surveyed in the range of 360–1000 nm wavelength using the spectrometer Ocean Optics QE 65000. It is worth noting that we did not observe any meaningful PL response to be higher than the noise level of the spectrometer within the examined spectral range (see the Supplementary, Figures S4 and S5). This confirms a low efficiency of radiative recombination of laser-light-injected charge carriers. In particular, the probability of radiative electron-hole recombination is very low due to the indirect character of the inter-band transition in anatase, and radiative recombination due to exciton annihilation usually observed at low temperatures is presumably suppressed in our case (probably because of the fast exciton dissociation to e^−^ and h^+^-polarons). This mechanism was discussed in Reference [35].

An important question relates to a probable influence of the laser-light-induced thermal effect on our measurements of the dc conductance in aTNTA. Actually, the laser heating of aTNTA under “hard” irradiation conditions can presumably cause even aTNTA structure modification (sintering, phase transformation) in the course of long-term irradiation. However, the effect of laser heating of the wide-zone semiconductor nanoparticles should be significantly less pronounced being compared with the laser heating of metal or carbon nanoparticles. In the case of aTNTA, efficiency of transformation of the electromagnetic field energy into internal energy of nanotubes is less due to the consumption of a significant part of electromagnetic energy in the inter-band transition. Rough estimates of the temperature increment in a single nanotube due to the action of a single laser pulse under the maximum energy of laser pulses, which we employed in our measurements, allows us to conclude that the heating effect of laser irradiation is rather subtle (see the Supplementary, Figure S2). Note that this issue was also discussed in Reference [36]. Direct measurements of the temperature of the aTNTA upper surface in the course of long-term irradiation using a high-sensitive thermal imaging system showed the absence of the laser-induced thermal effect (see the Supplementary, Figure S3). Additionally, experimentally observed after-irradiation restoration of the dc conductance of aTNTA to its initial value, even under long-term hard-mode action of pulse sequences, can be considered as indirect confirmation of the absence of thermally-induced structure changes.

## 3. Interpretation of Experimental Results

### 3.1. Kinetics of DC Conductance Decay in aTNTA

**Figure 4:**
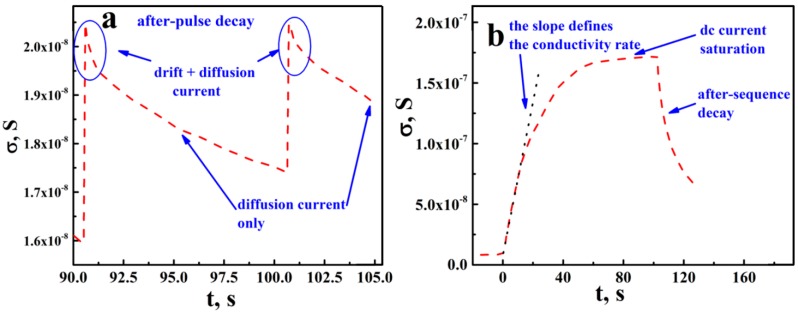
Examples of the decay kinetics in the photo-induced dc conductance of aTNTA; (**a**) a short-term (after-pulse) decay; (**b**) a long-term (after-sequence) decay; the red dashed lines correspond to the smoothed time-dependent data on the dc conductance; (**a**) the repetition rate is 0.1 Hz; the irradiation energy fluence is about 3.5 mJ/cm^2^; the initial stage of photo-activation; (**b**) the repetition rate is 2 Hz; irradiation energy fluence is about 3.5 mJ/cm^2^; duration of the pulse sequence is 103 s.

Figure 4 displays typical dc responses of the analyzed segment of aTNTA at short- (a) and long-term (b) scales. The conductance jumps observed in Figure 4a correspond to the periodic action of laser pulses. We have presumably interpreted an after-pulse decay of dc conductance (see Figure 4a) as a result of joint influence of the external-field driven and diffusion mechanisms for the long-range transport of mobile carriers throughout the aTNTA confined between electrodes. In particular, we assume a superposition of the drift and diffusion components at short time scales after the action of the laser pulse. With long time scales, only the diffusion transport of mobile carriers takes place; for more detailed arguments supporting this concept, see the Discussion section.

A decay of the dc conductance σ under the diffusion mode is mainly governed by a charge recombination in the aTNTA to be described by the kinetic equation dσ/dt=f(σ). A thorough analysis of the experimental data allowed us to assume the following form for this equation

(1) $$\frac{d\sigma }{dt}\approx -K\sigma^{\xi },\;\gamma >1,$$

where *K* denotes the decay rate constant. Under appropriately large values of the excess dc conductance (σ≥ 2 × 10^−8^ S), Equation (1) provides a reasonable fit of experimental data in the cases of a diffusion-controlled after-pulse decay (Figure 4a), as well as a long-term after-sequence decay (Figure 4b).

Indeed, the solution to Equation (1) with the initial condition $\sigma_{t=0}=\sigma_{0}$ leads to the following relationship

(2) $$\Lambda ={\left[\sigma \left(t^{\prime }\right)\right]}^{1-\xi }-\sigma_{0}^{1-\xi }\approx \left(\xi -1\right)Kt^{\prime }.$$

**Figure 5:**
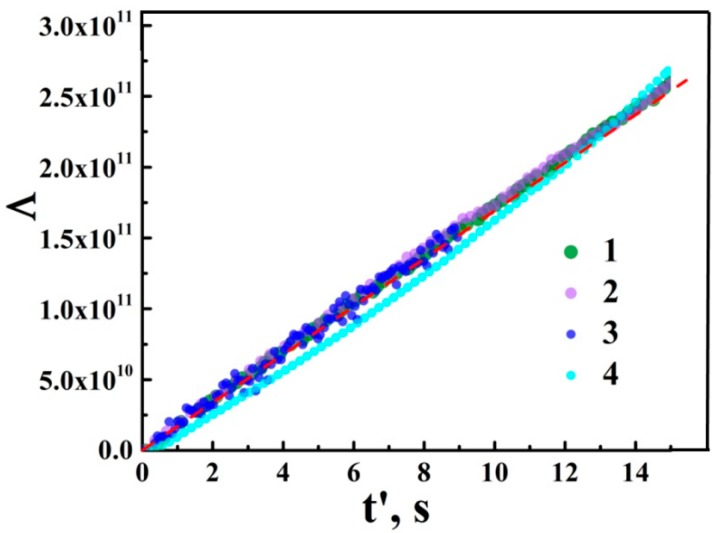
The recovered values Λ (the dimension of Λ is S^1−ξ^) against the time lapse; (1) an after-sequence decay; irradiation conditions: the wavelength is 355 nm; the irradiation fluence is 3.5 mJ/cm^2^; the repetition rate is 2 Hz; (2) an after-sequence decay; irradiation conditions: the wavelength is 355 nm; the irradiation fluence is 3.5 mJ/cm^2^; the repetition rate is 2 Hz; (3) an after-pulse decay; irradiation conditions: the wavelength is 355 nm; the irradiation fluence is 3.5 mJ/cm^2^; the repetition rate is 0.1 Hz; (4) an after-sequence decay; irradiation conditions: the wavelength is 355 nm; the irradiation fluence is 4 mJ/cm^2^; the repetition rate is 10 Hz. The red dashed line is displayed as a guide for the eye and corresponds to the linear dependence Λ on the time lapse retrieved with ξ≈ 2.62 and K≈ 1.7 × 10^10^ S^1−ξ^/s.

Here, $t^{\prime }$ is the time lapse after the action of the laser pulse or pulse sequence. Figure 5 displays the selectively chosen $\Lambda -t^{\prime }$ plots recovered using the experimental data for various irradiation and decay conditions. The dashed red line drawn as a guide for the eye corresponds to the decay rate constant K≈ 1.7 × 10^10^ S^1−ξ^/s and the exponent γ≈ 2.62. For the above mentioned, appropriately large values of dc conductance in the measured segment of aTNTA, the K and α values corresponding to the reasonable fitting accuracy (<15%) in an appropriately wide range of time lapse values (0 $<t^{\prime }\leq$ 100 s) are within the ranges 1.6 × 10^10^ S^1−ξ^/s ≤K≤ 1.8 × 10^10^ S^1−ξ^/s and 2.57 ≤γ≤ 2.65. Note that the decay exponent ξ has a fundamental relevance and is directly related to the dominating mechanism of the recombination of charge carriers; see a more detailed consideration in the Discussion.

### 3.2. The Quantum Yield of Photo-Activation and Mobility of Charge Carriers in aTNTA

Numerical integration of the “tails” of experimentally obtained dependencies σ(t) over the time intervals corresponding to restoration of the initial level of the aTNTA dc conductance (about 1.5 × 10^−8^ S, before the irradiation) under the single-pulse irradiation and pulse sequence irradiation allows us to estimate an excess photo-generated charge. Note that the characteristic restoration time lapse values for the dc conductance are of the order of several tens of seconds in the case of single-pulse irradiation and 1500 s–3000 s in the case of the pulse sequence irradiation.

With the obtained values of the dc conductance and the applied driven voltage (5 V), this corresponds to relatively large values of the photo-generated excess charge $Q_{ex}$ (from about 10^−6^ C for the single pulse activation to 10^−4^ C–10^−3^ C in case of the pulse sequence activation). Application of the conventional approach to the estimation of mobility parameters of excess charge carriers leads to extremely small values of these parameters. Indeed, we can write the following approximating expressions in the case of the dc-field-controlled drift transport

(3) $$\bar{j_{0}}\approx \frac{I_{0}}{S_{el}f}\approx e\bar{\mu_{c}}\bar{n_{c}}E_{ext}\approx \mu_{c}\frac{Q_{ex}}{V_{g}f}\cdot \frac{U}{d},$$

where $\bar{j_{0}}$ is the average current density at the initial stage of conductance decay, $I_{0}$ is the corresponding current value ($I_{0}=U\sigma_{t^{\prime }=0}$), U is the applied voltage, $S_{el}$ is the electrode surface area, f is the filling factor of the aTNTA, $\bar{\mu_{c}}$ and $\bar{n_{c}}$ are the average mobility and concentration of charge carriers, $E_{ext}$ is the external electrostatic field, $V_{g}$ is the geometric volume of the examined section, and d is the distance between the electrodes. Substituting the estimated values of the above listed parameters, we obtain the average mobility $\bar{\mu_{c}}$ to be about 10^−8^ cm^2^/Vs. In addition, assuming the diffusion mode of excess charge leakage from the examined section, we can write

(4) $$\bar{j_{0}}\approx \frac{I_{0}}{S_{el}f}\approx e\bar{D_{c}}\bar{grad\;n_{c}}\approx e\bar{D_{c}}\frac{Q_{ex}}{V_{g}fd},$$

where $\bar{D_{c}}$ is the average diffusion coefficient of charge carriers and $\bar{grad\;n_{c}}$ is the average gradient of the carrier concentration. Similarly to the previous case, we get an extremely low estimation of $\bar{D_{c}}$: ≈(10^−8^ ÷ 10^−7^) cm^2^/s. The obtained values of $\bar{\mu_{c}}$ and $\bar{D_{c}}$ are dramatically small in comparison with the values commonly reported for the anatase nanoparticles (see, e.g., Reference [10], where the electron mobility is 1–2 cm^2^/Vs). However, we must keep in mind that the reported data correspond to the microwave conductivity of closely packed ensembles of anatase nanoparticles and are related to the intraparticle (in our case, intra-tube) conductivity. Note that the results relating to the time-of-flight measurements of electronic conductivity in the nanoporous anatase ($\mu_{e}\approx$ 7 × 10^−6^ cm^2^/Vs, [8]) are the closest to our estimates. Here, the inter-tubular charge transfer should cause a giant influence on the long-range charge transport across the examined material. In addition, we can assume the occurrence of some mechanisms partially or almost totally blocking this transport; these issues are considered in the Discussion.

The estimated values of the excess photo-generated charge in the case of the single pulse action allows us to find the quantum yield of charge photo-generation. In particular, the photon fluence corresponding to the irradiation energy fluence $\Phi_{E}\approx$ 3.5 mJ/cm^2^ is approximately equal to the photon fluence $\Phi_{p}\approx \Phi_{E}\lambda /hc\approx$ 6.26 × 10^15^ cm^−2^ at λ= 355 nm. The absorption coefficient α of the anatase at 355 nm can be estimated as α=4πk/λ, where the optical constant k approximately equals 0.45. Thus, we can conclude that the following condition takes place: $\bar{h}>>\alpha^{-1}$, where $\bar{h}$ is the average height of the anatase nanotubes. Consequently, the incident light with λ= 355 nm is totally absorbed by the anatase component of the aTNTA, and we can estimate the number of absorbed photons for the examined aTNTA area *S_s_* per single laser pulse as $N_{p}=\Phi_{p}\cdot S_{s}\cdot f\approx$ 4.51 × 10^13^. On the other hand, estimations of excess charge in the material occurred due to irradiation by a single laser pulse give $Q_{ex}\approx$ (1.51 ± 0.48) × 10^−6^ C, or a number of excess carriers $N_{c}$ = (0.94 ± 0.30) × 10^13^. Accounting for these values, we can finally obtain the quantum yield of photo-activation of excess carriers as $\eta =N_{c}/N_{p}\approx$ 0.21 ± 0.07. The obtained value seems reasonable and is not very large due to detuning of the laser wavelength from the wavelength of the anatase maximum absorption.

The measured values of the dc conductance in the aTNTA tend to saturate at long durations of pulse sequences (Figure 4b) with a slow decay in the saturation level at large irradiation times (Figure 3a; this feature is highlighted by the dashed arrow). This slow decay is presumably associated with a gradual trapping of mobile carriers by the surface and bulk deep traps. In the case of “soft” and intermediate irradiation modes (see curve 1 in Figure 3a, and curves 1, 2 in Figure 3b), the saturation level goes up with an increasing repetition rate and energy of laser pulses. With the transition to “hard” irradiation modes (large values of the repetition rate and pulse energy, see curves 2–4 in Figure 3a, and curve 3 in Figure 3b), the saturation level asymptotically approaches a certain supreme value, which is independent from the repetition rate and pulse energy, and presumably indicates decay in the rate of inter-band transitions; see the Discussion. This supreme value of the saturation level has been evaluated for the analyzed segment of the aTNTA as about 7.0 × 10^−7^ S.

### 3.3. Photo-Activated DC Conductance of aTNTA Outside the Fundamental Absorption Band

One of the key characteristics of the charge transfer in semiconductors with the defects is based on estimation of the Urbach energy $E_{U}$ for the examined material. The $E_{U}$ value relates directly to such attributes of the energy band structure as the so-called steepness parameter g (see, e.g., [37]), which defines the efficiency of interaction of free and coupled charges with phonons. An increase in g causes an increasing probability of phonon-coupled charge states and their self-trapping. Therefore, the carrier mobility and efficiency of the charge transfer in the semiconductor material correlates with its Urbach energy.

**Figure 6:**
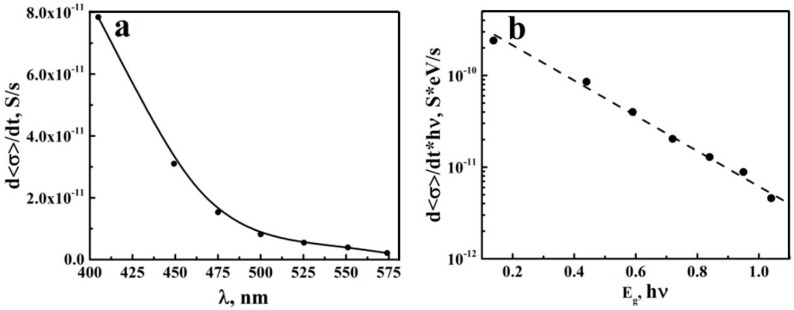
(**a**) the dependence of $E_{pt}\cdot d\langle \sigma \rangle /dt$ on the wavelength; (**b**) the Urbach plot for the examined segment of aTNTA. The points are experimental data, the curves are given as guides for the eye.

We used the obtained estimations of the average conductivity rate d〈σ〉/dt at the initial stage of photo-activation with various wavelengths of the laser light under the fixed irradiation fluence $\Phi_{E}\approx$ 3.5 mJ/cm^2^ and pulse repetition rate (10 Hz) (Figure 6a) for evaluation of $E_{U}$ in the examined system. The given evaluation is based on the following assumption: $d\langle \sigma \rangle /dt\propto \Delta N_{oc}\propto \left(\Phi_{E}\lambda /hc\right)\cdot f\cdot \left\{1-\exp\left[-\alpha \left(\lambda \right)\bar{h}\right]\right\}$, where $\Delta N_{oc}$ is the number of mobile charge carriers generated per unit of time and per unit square in the irradiated segment of aTNTA and $hc/\lambda =E_{pt}$ is the photon energy. The absorption coefficient α(λ) of the anatase outside the fundamental absorption band (for the wavelengths above approximately 387 nm) is rather small, and we can assume that the following condition takes place: $\alpha \left(\lambda \right)d_{ef}<<$ 1.

Consequently, we come to the following relationship $\alpha \left(E_{pt}\right)\propto E_{pt}\cdot d\langle \sigma \rangle /dt$. Figure 6b displays the values $E_{pt}\cdot d\langle \sigma \rangle /dt$ plotted against the difference of photon energy and bandgap energy of the anatase ($E_{pt}-E_{g}$) in the semi-logarithmic coordinates.

It is evident that a decay in $E_{pt}\cdot d\langle \sigma \rangle /dt$ with an increasing deviation in the photon energy from $E_{g}$ can be fitted with an appropriate accuracy by the exponential function

(5) $$\alpha \left(E_{pt}\right)\propto E_{pt}\cdot d\langle \sigma \rangle /dt\propto \exp\left[\left(E_{pt}-E_{g}\right)/E_{U}\right]$$

where the decay parameter $E_{U}$ associated with the Urbach energy approximately equals 240 meV. Note that this value significantly exceeds (at least 6–7 times) the values typical for the anatase single crystals at room temperature (see, e.g., Reference [38]).

On the other hand, the dependence of the absorption coefficient at the edge of the fundamental absorption band on the photon energy and absolute temperature can be expressed as [37]

(6) $$\alpha \left(E_{pt}\right)=\alpha_{0}\exp\left(g\frac{E_{pt}-E_{g}}{kT}\right)$$

where the above-mentioned steepness parameter g appears, which can be estimated to be approximately 0.105. The Urbach energy is inversely dependent on g at the fixed absolute temperature. The steepness parameter is the temperature-dependent value expressed as [37]

(7) $$g\left(T\right)=g_{0}\left(\frac{2kT}{E_{pn}}\right)\tanh\left(\frac{E_{pn}}{2kT}\right),$$

where $g_{0}$ is the supreme value of the steepness parameter at high temperatures, and $E_{pn}$ is the phonon energy. Following the estimations given in Reference 38, the temperature dependencies g(T) for the single-crystal anatase and rutile are close to saturation at the room temperature, and the corresponding values of the steepness parameter approach their supreme values. The effect of phonon-charge coupling can be quantified using the so-called coupling parameter, which is inversely proportional to $g_{0}$ or directly proportional to the Urbach energy. In particular, a large value of the coupling parameter for phonon-exciton interactions makes it possible to form the self-trapped excitons in anatase single crystals at the room temperature [38]. Note that the corresponding values of $g_{0}$ were estimated to be either around 0.733 or 0.621 in the dependence of orientation of the single crystal with respect to direction of the electric field in an incident light wave. Comparing these values with the estimated steepness parameter, we can expect sufficiently more pronounced coupling effects leading to significant suppression of the mobility of photogenerated charge carriers and the occurrence of the localized charge states.

### 3.4. A Recurrent Kinetic Model for Photo-Activated Evolution of DC Conductivity of aTNTA

Following the applied photo-activation technique, we may employ a recurrent kinetic model describing the temporal changes in the photo-induced dc conductance. Let $N_{c}^{\left(k\right)}$ be the number of mobile charge carriers of any type (electrons, holes, e- and h-type polarons) accumulated in the photo-activated nanotubular layer in the time of action of the k-th laser pulse. The population $N_{c}^{\left(k\right)}$ directly relates to the dc conductance in the examined aTNTA. Also, we denote a population of absorbing centers (coupled charge carriers) in the ground state (the valence band), which are ready for photoionization, as $N_{g}^{\left(k\right)}$. We can write the following system of equations corresponding to the initial stage of photo-activation (the action of the primary laser pulse)

(8) $$\left\{ \begin{array}{l} N_{c}^{\left(1\right)}=N_{g}^{\left(0\right)}\alpha \left(N_{c}^{\left(0\right)}\right)\left[1-\beta \left(N_{c}^{\left(0\right)}\right)-\gamma \left(N_{c}^{\left(0\right)}\right)\right];\\ N_{g}^{\left(1\right)}=N_{g}^{\left(0\right)}-N_{g}^{\left(0\right)}\alpha \left(N_{c}^{\left(0\right)}\right)\left[1-\beta \left(N_{c}^{\left(0\right)}\right)\right], \end{array}\right.$$

where the dimensionless efficiency factors α,β,γ of photoionization, charge recombination, and trapping, respectively, are assumed to be dependent in the common case on the irradiation conditions (the pulse energy (α) and the time interval between sequential pulses (β,γ)) and the population $N_{c}^{\left(k\right)}$. Accordingly, we obtain the following system of equations in case of an arbitrary number of acting laser pulses as

(9) $$\left\{ \begin{array}{l} N_{g}^{\left(k\right)}=N_{g}^{\left(0\right)}{\left\{1-\alpha \left(N_{c}^{\left(k-1\right)}\right)\left[1-\beta \left(N_{c}^{\left(k-1\right)}\right)\right]\right\}}^{k};\\ N_{c}^{\left(k\right)}=N_{g}^{\left(0\right)}\alpha \left(N_{c}^{\left(k-1\right)}\right)\left[\begin{array}{l} \left\{\left[1-\gamma \left(N_{c}^{\left(k-1\right)}\right)\right]+\left[1-\beta \left(N_{c}^{\left(k-1\right)}\right)\right]\left[1-\alpha \left(N_{c}^{\left(k-1\right)}\right)\right]\right\}\times \\ \times {\left[1-\beta \left(N_{c}^{\left(k-1\right)}\right)-\gamma \left(N_{c}^{\left(k-1\right)}\right)\right]}^{k-1}+\\ +\sum_{m=2}^{k-1}{\left\{1-\alpha \left(N_{c}^{\left(k-1\right)}\right)\left[1-\beta \left(N_{c}^{\left(k-1\right)}\right)\right]\right\}}^{m}{\left[1-\beta \left(N_{c}^{\left(k-1\right)}\right)-\gamma \left(N_{c}^{\left(k-1\right)}\right)\right]}^{k-m} \end{array}\right]. \end{array}\right.$$

It should be noted that the assumed model has a sufficiently more generalized form compared to other kinetic models applied for a detailed description of photoinduced charge transfer in semiconductors (see, e.g., Reference [39]). In particular, the factor β considers all contributions of various radiative and non-radiative recombination mechanisms leading to restoration of the population of absorbing centers ready for the follow-up photoionization during the action of the next laser pulse. Similarly, the trapping factor γ is related to various charge carriers (electrons, holes, e-type and h-type polarons) which irretrievably transit from the free (mobile) state to the arrested state. Despite its general character, this model can be applied to analyze the observed peculiarities in the behavior of photo-activated conductance for the examined aTNTA by taking into account physically reasonable factors.

The important item relates to the dependence of the photo-ionization factor α on the concentration of mobile carriers. In the case of photo-activation with the photon energies insignificantly exceeding the bandgap of the examined structure, the long-term action of pulse sequences under low efficiency of recombination and trapping channels will lead to an increasing occupation of the near-bottom region in the aTNTA conduction band by mobile electrons and e-type polarons. As a result, the quantum efficiency of the inter-band transition must gradually decrease with the increasing population $N_{c}^{\left(k\right)}$. In our opinion, this effect combined with a partial depletion in the $N_{g}^{\left(k\right)}$ population is the reason for an experimentally observed saturation of the photo-activated dc conductance up to extreme level (Figure 3a,b).

Using the considered model, we have carried out a pilot modeling of the photo-induced response under the following assumptions:

- The photo-induced conductance of the examined aTNTA is proportional to the current population $N_{c}^{\left(k\right)}$;
- the time lapse t after beginning the photoactivation process is defined as $t\approx k/\nu_{r}$, where $\nu_{r}$ is the repetition rate of laser pulses;
- the influence of the population $N_{c}^{\left(k\right)}$ on the photo-ionization efficiency α is assumed to be a monotonically decaying function of $N_{c}^{\left(k\right)}$; we have assumed a linear decay in α described by the following relationship
  (10) $$\left\{ \begin{array}{l} \alpha \left(N_{c}^{\left(k\right)}\right)=\alpha_{0}\left(1-\frac{N_{c}^{\left(k\right)}}{N_{c}^{crit}}\right),N_{c}^{\left(k\right)}\leq N_{c}^{crit};\\ \alpha \left(N_{c}^{\left(k\right)}\right)=0,N_{c}^{\left(k\right)}>N_{c}^{crit}; \end{array}\right.$$
  where the critical population $N_{c}^{crit}$ is assumed equal to $N_{g}^{\left(0\right)}/10$;
- the recombination efficiency β and the trapping efficiency γ are assumed to be independent from the population $N_{c}^{\left(k\right)}$.

**Figure 7:**
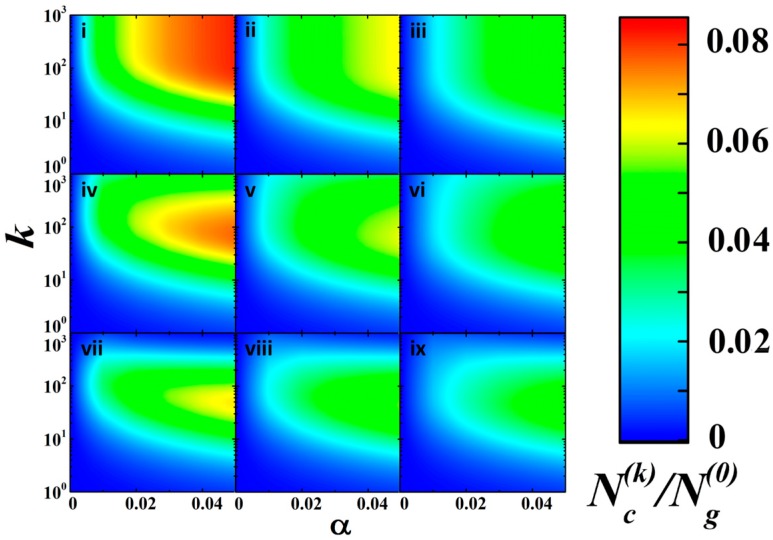
2D plots of the normalized population $N_{c}^{\left(k\right)}/N_{g}^{\left(0\right)}$ against the number of acting laser pulses and the initial efficiency of photo-ionization for the various values of β and γ. (**i**) β = 0.01, γ = 0; (**ii**) β = 0.025, γ = 0; (**iii**) β = 0.04, γ = 0; (**iv**) β = 0.01, γ = 10^−3^; (**v**) β = 0.025, γ = 10^−3^; (**vi**) β = 0.04, γ = 10^−3^; (**vii**) β = 0.01, γ = 5 × 10^−3^; (**viii**) β = 0.025, γ = 5 × 10^−3^; (**ix**) β = 0.04, γ = 5 × 10^−3^.

The simulation results are shown in Figure 7 for the various relationships between the model parameters as 2D plots of the normalized population $N_{c}^{\left(k\right)}/N_{g}^{\left(0\right)}$.

It should be noted that despite greatly simplified and sometimes debatable assumptions (a linear decay in α with a gradual increase in the population $N_{c}^{\left(k\right)}$, equality of the critical population to $N_{g}^{\left(0\right)}/10$, independence of β and γ from $N_{c}^{\left(k\right)}$), the modeling results are qualitatively consistent with experimentally observed tendencies in the behavior of the photo-induced conductance. In particular, the presented 2D plots reflect the basic features in this behavior, i.e., the effect of saturation and existence of the extreme saturation level, the influence of irradiation fluence (via the photo-ionization efficiency α) and the recombination rate (via the recombination efficiency β), and occurrence of a slow decay in the saturation level with the increasing trapping efficiency γ. Comparing the modeled data with experimentally observed tendencies in the long-term behavior of the dc conductance of the aTNTA (in the part of a relatively fast saturation with further slow decay, see Figure 3a), we can arrive at the following reasonable relationships between the model rate parameters α,β, and γ:γ≈ (1 ± 0.3) × 10^−3^β; β≈ (0.71 ± 0.25)α; these limitations correspond to the panels v-vi in Figure 7. The future efforts will be directed to a more precise adaptation of the developed model to the examined system.

## 4. Discussion

Based on the obtained experimental data, we can single out such features of the long-range charge transport in the examined aTNTAs as extremely low values of charge mobility at the room temperature, saturation of the dc conduction under a long-term action of laser pulses, and large value of the Urbach energy.

**Figure 8:**
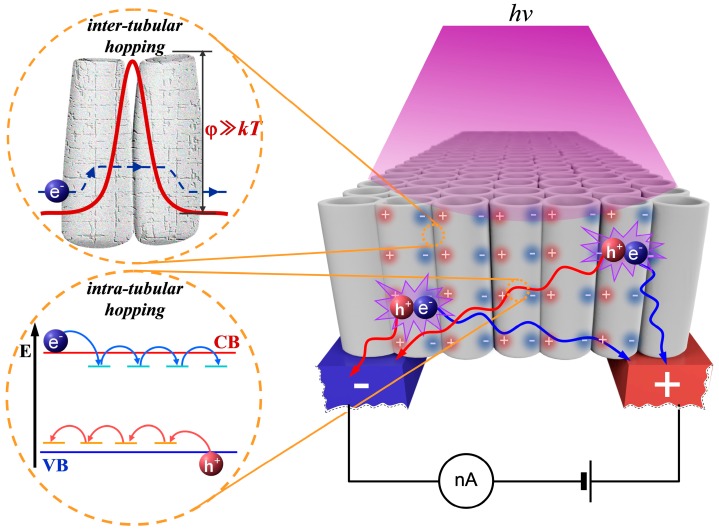
The cartoon displaying electrical processes in aTNTA under laser irradiation.

A surprisingly low value of the charge mobility μ recovered from the measurements of the photo-activated dc conductance contradicts the abundance of reported experimental results on μ values for the anatase nanoparticles. Typically, these reports give the values of charge mobility ranging from 0.01 cm^2^/Vs to 10 cm^2^/Vs. However, we should keep in mind that experimental techniques used in these studies are based on contactless microwave or Terahertz technologies. These approaches are able to characterize only the “local” average mobility of charge carriers in the vicinity of their current positions due to the oscillating character of the probe electromagnetic field. Indeed, the expected displacement of the carriers over half of the oscillation period at the frequencies 10^10^–10^11^ Hz does not exceed several nanometers even in the case of large fluence rates of probing electromagnetic radiation. In other words, an arbitrarily chosen charge carrier cannot leave its local habit zone and travel along the assembly of nanoparticles in the course of microwave or Terahertz probes. On the contrary, in our case, the long-path-travelling carriers propagating over distances of the order of several tens of micrometers play a major role in the measured dc conductance of the aTNTA. From this point of view, our results can be compared with the long-standing experimental data on charge mobility in the nanoporous anatase, which were obtained using the time-of-flight technique [8]. We can assume that the obtained small μ values are caused by the sequential acts of hopping charge transfer between the adjacent anatase nanotubes as we can see in Figure 8.

A similar charge transfer mechanism in the clusters of anatase nanoparticles was discussed by Fravventura et al. [10,11]. They concluded that despite the relatively large “intra-particle” values of the charge mobility measured using the microwave technique, the overall cluster conductivity is strongly limited by a large trap density reducing the population of conducting band electrons and thermally activated hops between the adjacent nanoparticles. In combination with the above mentioned strong charge-phonon coupling leading to prevailing polaronic conductivity in the nanostructured anatase, the hopping “inter-tubular” charge transfer in the examined array causes low-efficiency macroscopic long-range transport of the mobile carriers.

A detailed quantitative analysis of the overall transport mechanism that appears in such structures can be addressed to principles of the percolation theory (see, e.g., References [40,41]). In the framework of this approach, the array of anatase nanotubes can be considered as a low-conducting 2D network with energy barriers in the nodes and predominating hopping conductivity of the node connections [42,43]. This issue will be the subject for future detailed studies.

The experimentally observed power-law decay in the dc conductance of the aTNTA is evidently correlated with a decay in the population of mobile carriers caused by the sequences of recombination acts. The obtained value of the decay exponent ξ≈ 2.6 can be compared with the similar values discussed in the literature. In particular, the simplest description of charge carrier recombination in the framework of Langevin’s model referred to many times is based on consideration of the Coulomb interaction between positive and negative carriers. The Langevin’s model predicts a carrier concentration decay to be inversely proportional to the time lapse, $N_{c}\sim {t^{\prime }}^{-1}$. However, numerous studies of carrier recombination in the various photovoltaic devices show significant deviations from this model; typically, these units exhibit a power-law decay in the recombination rate described as $dN_{c}/dt^{\prime }\sim {N_{c}}^{1+\vartheta }$ (see, e.g., Reference [44]) with ϑ in the range from 1.5 to 2.5 dependent upon the technology of the unit fabrication. Note that our value of the decay exponent is close to the lower limit in the given range.

Another important issue is related to a possible blocking of the driving action of the external electrostatic field $E_{ext}$ due to separation of the positive and negative carriers. Because of high concentrations of photoinduced excess carriers, small spatial scales of the irradiated system and low rates of the carrier recombination, their separation under the action of the external field associated with the applied voltage should lead to the appearance of the internal confining field $E_{con}$ in the aTNTA structure, which is directed opposite to the external field. As a result, a quasi-equilibrium carrier distribution occurs in the aTNTA and the drift component of the dc current is almost totally suppressed. Thus, the dc response of the aTNTA is defined primarily by the diffusion current. Taking into account the slow character of the charge transport between the adjacent nanotubes due to high barriers, we can assume the formation of these spatially separated quasi-equilibrium carrier distributions in each nanotube. In this case, short-term disturbances of such quasi-equilibrium states due to photoinduced injections of excess carriers will fade; we can see manifestation of this relaxation in Figure 4a marked as after-pulse conductance drops. The drop duration is of the order of 0.1–0.3 s and is presumably associated with the time of the carrier travel over the distance of the order of the nanotube diameter. Respectively, the average travel velocity $\bar{v}$ is of the order of 10^−4^ cm/s. With the vanishing driving field $\Delta E=E_{ext}-E_{con}$ the average intra-tubular mobility of charge carriers $\bar{\mu }=\bar{v}/\Delta E$ can be comparable with the usually reported microwave data on the carrier mobility for the anatase nanoparticles. The discussed details of the macroscopic and microscopic carrier transport are illustrated in Figure 8.

It should be noted that under relatively low values of repetition rate of laser irradiation and pulse energy (the above mentioned “soft” and intermediate irradiation modes) as well as a low recombination rate, the saturation of aTNTA conductance is controlled by the following stationary condition $\Delta N_{i}=\Delta N_{r}$, where $\Delta N_{i}$ is the number of mobile carriers injected during the action of the laser pulse, and $\Delta N_{r}$ is the number of carriers recombining during the time interval between the sequential laser pulses. In the framework of the above considered recurrent kinetic model, the value $\Delta N_{i}$ can be defined as $\Delta N_{i}=\alpha N_{g}\approx \eta \left(\Phi_{E}S_{s}f\lambda /hc\right)N_{g}$, where $N_{g}$ is the current population of the ground state. Assuming the above discussed power-law recombination decay, the number of recombining carriers can be expressed as

(11) $$\Delta N_{r}=\overset{1/\nu_{r}}{\underset{0}{\int }}\left(\frac{dN_{c}}{dt}\right)dt\approx \frac{KN_{c}^{\xi }}{\nu_{r}},$$

where $N_{c}$ is the current population of the mobile carriers and $\nu_{r}$ is the repetition rate of laser pulses. Thus, we can conclude that the saturation level for $N_{c}^{sat}$ changes depending on irradiation parameters as $N_{c}^{sat}\sim {\left(\Phi_{E}v_{r}\right)}^{1/\xi }$. This conclusion is qualitatively consistent with experimentally observed tendencies in the behavior of the photoinduced dc conductance in the aTNTA (Figure 3a,b) and the simulation results (Figure 7). The transition from the intermediate to “hard” irradiation modes, due to a further increase in the $\Phi_{E}$ and $v_{r}$ values, should lead to the above mentioned suppression of efficiency of the inter-band transition (the decay in α) and we arrive at a totally saturated system with very slowly decaying conductance due to irreversible trapping of the mobile carriers. 

## 5. Conclusions

The results obtained in this study allow us to highlight the following features of long-range transport of charge carriers throughout the aTNTAs. The estimated large values of the Urbach energy indicate quite strong coupling of charge carriers with phonons in this material which means that the photo-induced conductivity is matured from the polaronic type, rather than from the conventional transfer of free electrons and holes. Therefore, the probability of occurrence of the self-trapped charge states is adequately high. The major factor to control the macroscopic dc conductivity is the inter-tubular charge transfer which has a hopping character due to relatively high barriers at the interfaces between the adjacent nanotubes. These interfaces act like “bottlenecks” in the overall long-range transport of carriers. Separation of the accumulated positive and negative mobile carriers due to the action of the external electrostatic field results in the appearance of the counteracting internal field within the nanotubes and almost total suppression of the drift current inside the structure. Therefore, the leakage of accumulated carriers into the external circuit is governed by the diffusion transfer of carriers. These peculiarities cause an extremely low value of macroscopic (average) mobility of charge carriers when compared to the usually reported intra-particle mobility for the nanostructured anatase. The reported results should be taken into account when developing different kinds of electronic units based on the aTNTAs, which deal with the generation of free carries due to external inputs as the discussed light action or even a gas media. We hope to extend our findings later to the gas sensor phenomenon.

## Supplementary Materials

The following are available online at http://www.mdpi.com/2079-4991/8/11/915/s1. Figure S1: The electron microscopy imaging of aTNTAs: (a) SEM photo of the cross-section of the array; (b) HR-TEM image of surface at the wall of a single titanium dioxide nanotube, Figure S2: The possible thermal heating effect on the single TiO_2_ nanotube: the time of laser irradiation (*t ≥ τ_i)_*; the time of thermal relaxation just after the laser irradiation (*t ≈ Δt_T_)*; time after relaxation of thermal effect prior the next laser pulse irradiation (*Δt_T_ < t)*, Figure S3: The IR images of the the multielectrode chip equipped with aTNTA: (a) the original IR images of the chip: top—before laser irradiation, down—immediately after laser irradiation, (b) the differential image built by subtraction of the IR images recorded before and immediately after laser irradiation, Figure S4: The experimental setup used to observe the possible photoluminescence response of the laser-irradiated aTNTA, Figure S5: Back-reflectance spectra of the laser-irradiated aTNTA (2) and reflectance standard (3). 1—the “dark” spectrum (in the absence of laser irradiation). The peaks ranging from 300 nm to 400 nm correspond to the acting laser radiation at 355 nm (broadening of the observed laser line is caused by the imperfect modal structure of the laser beam, oversaturation of the pixels of the spectrometer sensor, etc.). Higher intensity of the laser peak in the case of the reflectance standard is obviously caused by higher reflectivity of this sample.
