# Supplementary material for: Light-Tuned DC Conductance of Anatase TiO2 Nanotubular Arrays: Features of Long-Range Charge Transport
Source: Nanomaterials (Basel). 2018 Nov 7;8(11):915. doi: 10.3390/nano8110915 (PMC6266825; doi:10.3390/nano8110915)
Supplement: Supplementary file 1 [file nanomaterials-08-00915-s001.pdf]

nanomaterials

MDPI

Supplementary Materials "Light-Tuned DC Conductance of Anatase TiO_2 Nanotubular Arrays: Features of Long-Range Charge Transport"

Dmitry A. Zimnyakov*[1,2]*, Michail Yu. Vasilkov[1,3], Sergey A. Yuvchenko[1], Alexey S. Varezhnikov[1], Martin Sommer[4], and Victor V. Sysoev[1]

[1] Physics Department, Yuri Gagarin State Technical University of Saratov, 77 Polytechnicheskaya str., Saratov 410054, Russia; vasilk.mikhail@yandex.ru (M.Yu.V.); yuv-sergej@yandex.ru (S.A.Yu.); alexspb88@mail.ru (A.S.V.); vsysoev@sstu.ru (V.V.S.)
[2] Precision Mechanics and Control Institute of Russian Academy of Sciences, 24 Rabochaya str., Saratov 410024, Russia
[3] Saratov Branch of Kotelnikov Institute of Radio-Engineering and Electronics of Russian Academy of Sciences, 38 Zelenaya str., Saratov 410019, Russia
[4] Institute of Microstructure Technology, Karlsruhe Institute of Technology, 1 Hermann-von-Helmholtz Platz, 76344 Eggenstein-Leopoldshafen, Germany; martin.sommer@kit.edu
* Correspondence: zimnykov@mail.ru
Tel.: +7-8452-998624

1. Electron microscopy imaging of aTNTAs (Section 2.1)

The thorough characterization of the aTNTAs by scanning and transmission electron microscopy (SEM, TEM) and X-ray photoelectron spectroscopy has been performed elsewhere [S1, ref. 27 of main text]. The Figure S1 gives the SEM/TEM overview.

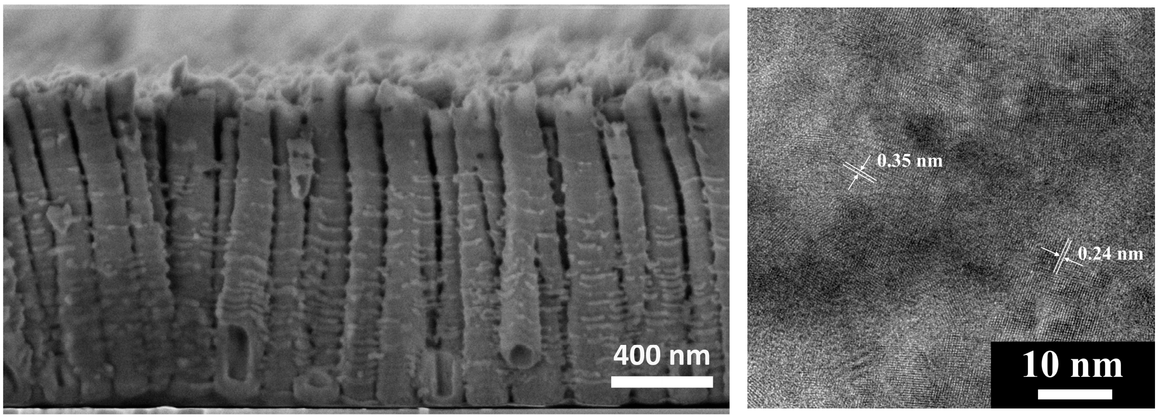

(a) (b)

Figure S1. The electron microscopy imaging of aTNTAs: a) SEM photo of the cross-section of the array; b) HR-TEM image of surface at the wall of a single titanium dioxide nanotube.

2. The thermal effect under laser irradiation of aTNTA (Section 2.2)

2.1. The theoretical approach

Let us consider the laser-pulse-caused thermal effect in a single anatase nanotube using the adiabatic approximation. In the framework of current understanding of the light-matter interaction, the increase in the internal energy of an irradiated object is caused by interactions of photoinduced "hot" mobile charge carriers with a lattice. In the case of inter-band transition, the kinetic energy of

mobile carriers (electrons) in the conduction band is defined by the difference between the photon energy and the bandgap energy. This causes low efficiency of transformation of the electromagnetic field energy to the internal energy of the nanotubes; a corresponding diminishing factor can be introduced as

$$\Upsilon = \frac{E_{ph} - E_g}{E_{ph}},$$

where E_{ph} is the photon energy and E_g is the bandgap energy. In our case the diminishing factor is approximately equal to 0.083.

In the framework of the adiabatic approximation, the following energy balance equation can be written (Fig. S2, $t \geq \tau_i$) as

$$\Upsilon \Phi_E \tilde{S} \approx C_p \tilde{V} \rho \Delta T \approx C_p \tilde{S} \alpha^{-1} \rho \Delta T,$$

where Φ_E is the irradiation energy fluence, $\tilde{S}$ is the geometric cross-section of the nanotube, C_p and ρ are the specific heat and density of anatase, $\tilde{V}$ is the irradiated volume of the nanotube, ΔT is the temperature increment after the action of a single laser pulse, and α is the absorption coefficient of anatase. Applying the physical constants for bulk anatase (the molar heat capacity is approx. 55 J/mol·K at room temperature [S2], the molar mass is approx. 79.87 kg/kmol, and the density is approx. $3.78 \cdot 10^3$ kg/m^3, and the absorption coefficient as $\alpha = 4\pi k/\lambda$, where k is the optical constant of anatase equal to about 0.40 at 355 nm [S3]), we can obtain finally $\Delta T \approx$ 10 - 13 K at $\Phi_E \approx$ 4.0 mJ/cm^2.

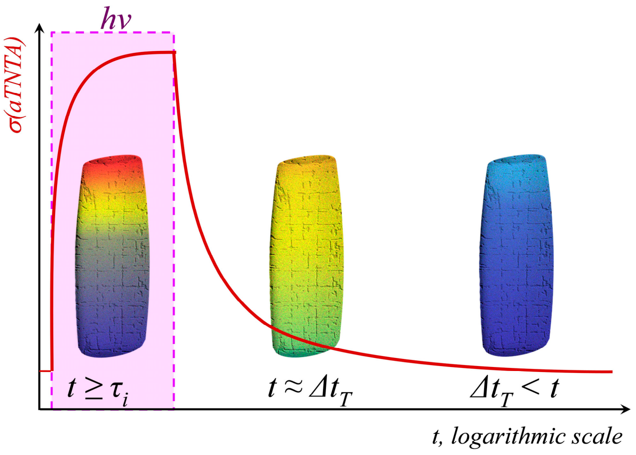

Figure S2. The possible thermal heating effect on the single TiO_2 nanotube: the time of laser irradiation ($t \geq \tau_{i);}$) the time of thermal relaxation just after the laser irradiation ($t \approx \Delta t_T$); time after relaxation of thermal effect prior the next laser pulse irradiation ($\Delta t_T < t$)

The kinetics of the after-pulse temperature relaxation in the nanotube due to the thermal conductivity is controlled by the thermal diffusivity of anatase $\tilde{a} = \lambda / C_p \rho$, where λ is the thermal conductivity. Using the anatase data taken from the literature, we can estimate $\tilde{a}$ as about $2.8 \cdot 10^{-6}$ m^2/s. The characteristic time of temperature equalization (Fig. S2, $t \approx \Delta t_T$) in the nanotube volume can be estimated as $\Delta t_T \propto h^2/\tilde{a}$, where h is the nanotube height. Rough estimates give the value Δt_T of the order of few microseconds (which is significantly smaller than the time interval between

sequential laser pulses even in the case of "hard" irradiation modes), and excess residual temperature in this case roughly estimated in the framework of the adiabatic approximation as $\Delta T\tilde{V}/V_{total}$ (V_{total} is the total volume of the nanotube) is small (of the order of 1 K). Therefore, we can expect the excess residual temperature falling to 0 during the time interval between the sequential pulses (Fig. S2, $\Delta t_T < t$). This conclusion is supported by the results of thermal imaging of the long-term-irradiated aTNTA (Fig. S3).

2.2. The IR camera measurements of multielectrode chip equipped with aTNTA

In order to estimate the thermal effect of laser irradiation on the aTNTA, we have performed IR camera (Testo 875) measurements of the surface of multielectrode chip with and without laser irradiation. Laser irradiation at 4 mJ/cm^2 power density, 10 Hz pulse frequency, has been applied for 30 min at the wavelength of 355 nm. Figure S3a displays the recorded IR images. The temperature difference (Fig. S3b) does not exceed one degree.

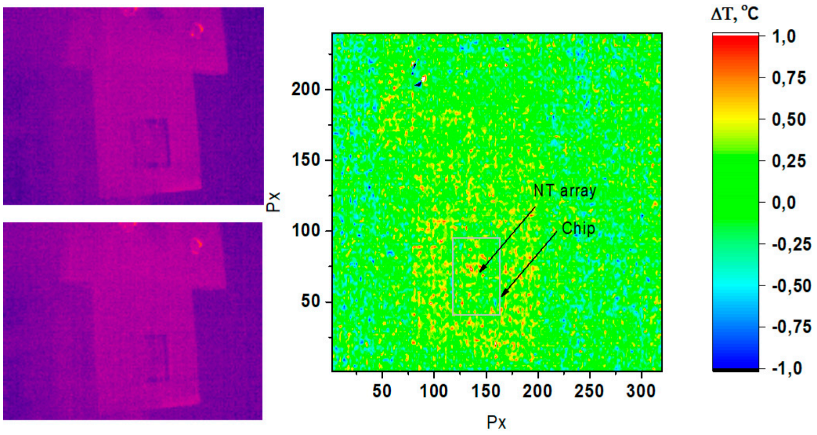

(a) **(b)**

Figure S3. The IR images of the the multielectrode chip equipped with aTNTA: a) the original IR images of the chip: top - before laser irradiation, down – immediately after laser irradiation, b) the differential image built by subtraction of the IR images recorded before and immediately after laser irradiation.

3. Analysis of the photoluminescence response of laser-irradiated aTNTA (Section 2.2)

Figure S4 shows the experimental setup used to verify the possible photoluminescence response of the laser-irradiated aTNTA. The light output from the laser-irradiated area was analyzed using the spectrometer Ocean Optics QE 65000. Light was collected by the fiber-optical probe connected with the entrance port of the spectrometer. We applied the "hard" irradiation mode (the irradiation fluence is 4mJ/cm^2 at 355 nm, and the repetition rate is 10 Hz). The integration time of the spectrometer was set at 10 s. For comparison, we also recorded the optical response from the laser-irradiated reflectance standard WS-1 PIXELTEQ (this sample undoubtedly does not have any photoluminescence response in the near UV, visible, or near IR regions). Irradiation conditions were the same for two examined samples, the aTNTA and the reflectance standard. The obtained spectra of the back-reflected light are displayed in Figure S5.

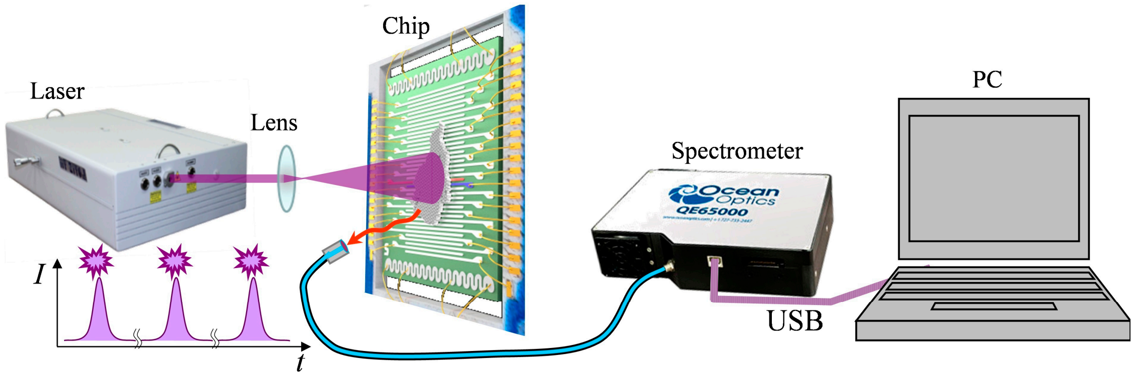

Figure S4. The experimental setup used to observe the possible photoluminescence response of the laser-irradiated aTNTA.

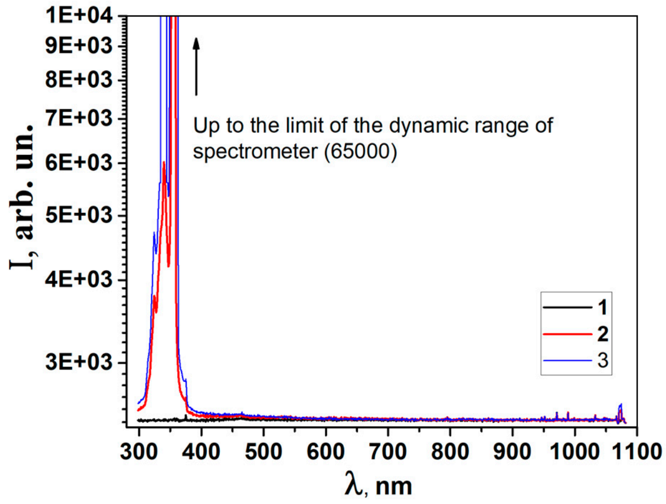

Figure S5. Back-reflectance spectra of the laser-irradiated aTNTA (2) and reflectance standard (3). 1 – the "dark" spectrum (in the absence of laser irradiation). The peaks ranging from 300 nm to 400 nm correspond to the acting laser radiation at 355 nm (broadening of the observed laser line is caused by the imperfect modal structure of the laser beam, oversaturation of the pixels of the spectrometer sensor, etc.). Higher intensity of the laser peak in the case of the reflectance standard is obviously caused by higher reflectivity of this sample.

Thus, comparison of the obtained spectra allows us to conclude that there is no photoluminescence response from the laser-irradiated aTNTA in the spectral range from 400 nm to 1100 nm.

References

S1. Fedorov, F.; Vasilkov, M.; Lashkov, A.; Varezhnikov, A.; Fuchs, D.; Kübel, Ch.; Bruns, M.; Sommer, M.; Sysoev, V. Toward new gas-analytical multisensor chips based on titanium oxide nanotube array. *Scientific Reports* **2017**, *7*, 9732. https://doi.org/10.1038/s41598-017-10495-8

S2. Chase, M.W., Jr. NIST-JANAF Themochemical Tables, Fourth Edition, *J. Phys. Chem. Ref. Data*, *Monograph 9*, **1998**, 1-1951.

S3. http://matprop.ru/img/nk/Oxides/tio2.gif.
